# Estimates of short sleep duration among US rural and urban women with prediabetes

**DOI:** 10.1371/journal.pone.0284304

**Published:** 2023-04-06

**Authors:** Phoebe Tran, Brittany Shelton, Liem Tran

**Affiliations:** 1 Department of Public Health, University of Tennessee, Knoxville, TN, United States of America; 2 Department of Geography & Sustainability, University of Tennessee, Knoxville, TN, United States of America; Shahid Beheshti University of Medical Sciences, ISLAMIC REPUBLIC OF IRAN

## Abstract

**Background:**

Short sleep duration (SSD) (<7 hours/night) is linked with increased risk of prediabetes to diabetes progression. Despite a high diabetes burden in US rural women, existing research does not provide SSD estimates for this population.

**Methods:**

We used national Behavioral Risk Factor Surveillance System surveys to conduct a cross-sectional study examining SSD estimates for US women with prediabetes by rural/urban residence between 2016–2020. We applied logistic regression models to the BRFSS dataset to ascertain associations between rural/urban residence status and SSD prior to and following adjustment for sociodemographic factors (age, race, education, income, health care coverage, having a personal doctor).

**Results:**

Our study included 20,997 women with prediabetes (33.7% rural). SSD prevalence was similar between rural (35.5%, 95% CI: 33.0%-38.0%) and urban women (35.4%, 95% CI: 33.7%-37.1). Rural residence was not associated with SSD among US women with prediabetes prior to adjustment (Odds Ratio: 1.00, 95% CI: 0.87–1.14) or following adjustment for sociodemographic factors (Adjusted Odds Ratio: 1.06, 95% CI: 0.92–1.22). Among women with prediabetes, irrespective of rural/urban residence status, being Black, aged <65 years, and earning <$50,000 was linked with significantly higher odds of having SSD.

**Conclusions:**

Despite the finding that SSD estimates among women with prediabetes did not vary by rural/urban residence status, 35% of rural women with prediabetes had SSD. Efforts to reduce diabetes burden in rural areas may benefit from incorporating strategies to improve sleep duration along with other known diabetes risk factors among rural women with prediabetes from certain sociodemographic backgrounds.

## Introduction

Approximately 47 million United States (US) women have prediabetes, an often asymptomatic condition characterized by having elevated blood sugar levels (hemoglobin A1C test: 5.7–6.4%) that do not yet meet the cutoff for diabetes (hemoglobin A1C test: ≥6.5%) [1]. An estimated 70% of prediabetes cases are expected to progress to diabetes [2, 3]. In addition to the clinical consequences of prediabetes and diabetes, these conditions respectively cost the US around $43.4 billion and $360.6 billion each year in terms of medical expenses and missed productivity [4]. While the burden of prediabetes and diabetes in the United States is notable, this very burden varies between sociodemographic groups and geographic areas [5–8].

One such example of a US diabetes burden disparity is that there is a 17% higher diabetes prevalence among rural populations as compared to urban populations [9–11]. This increased prevalence of diabetes may be attributable to the management of prediabetes in rural settings. For example, prediabetes cases among rural US women may be more likely to progress to diabetes due to greater obesity and physical inactivity rates in US rural areas [12]. Additionally, other modifiable factors may also contribute to this observed inequity. Like obesity and physical inactivity, short sleep duration (SSD) (<7 hours/night), for example, has also been linked with prediabetes to diabetes progression [13, 14]. A systematic review conducted across different populations, including South Korean, Finnish, and American individuals, indicated that SSD was associated with a 59% (95% CI: 29%-97%) increased risk of progression from prediabetes to diabetes [13]. Thus, understanding the prevalence of SSD among individuals with prediabetes is critical for the identification of modifiable risk factors for diabetes development.

Despite a high diabetes burden in US rural women and an association between SSD and progression to diabetes, there is little information on SSD estimates for US women in rural areas with prediabetes. Existing US research points towards high levels of short sleep duration (38.5%, 95% CI: 37.5%-39.6%) among rural residents nationwide, but this finding may not apply to rural women with prediabetes [15]. Additionally, the few US observational and interventional studies that have examined SSD among individuals with diabetes do not provide estimates specific to women or rural residents [16–18]. As such information can inform evidence-based diabetes prevention for women in medically underserved rural areas, we conducted an observational study using cross-sectional data from 2016–2020 to ascertain SSD estimates for US women with prediabetes by rural/urban residence status. We also determined if associations between rural/urban residence status and SSD were present prior to and following adjustment for prediabetes- and sleep-related sociodemographic factors.

## Methods

### Study data and variables

We used data from the 2016–2020 Behavioral Risk Factor Surveillance System (BRFSS) surveys except for 2019, as this survey year does not contain sleep duration information. The BRFSS is a nationally representative cross-sectional survey whose purpose is to collect information on US individuals’ sociodemographic background, existing health conditions, and health behaviors. The survey is administered annually via landline and cell phone by the Centers for Disease Control and Prevention (CDC) to noninstitutionalized US residents aged ≥18 years [19]. Survey weights and oversampling of US groups that are underrepresented in certain parts of the country (i.e., racial/ethnic minorities, rural residents) are used to ensure representativeness of BRFSS estimates.

From the BRFSS surveys, we obtained information on sleep duration, prediabetes status, and rural/urban residence. In keeping with CDC guidelines, individuals whose response was <7 hours of sleep/night to the BRFSS question, “On average, how many hours of sleep do you get in a 24-hour period?” were considered to have SSD [20–25]. BRFSS metropolitan status codes were used to categorize survey participants into rural and urban residents. Data on sociodemographic factors (age, sex, race, education, income, health care coverage, having a personal doctor) consistent with prior US SSD research was also extracted from the BRFSS [16–18].

### Ethics

Ethics approval and informed consent were not sought for this study due to BRFSS data falling under exemption status for IRB review at the authors’ institution. BRFSS surveys have undergone the CDC’s IRB and informed consent process such that all survey respondents provide informed consent. Additionally, only deidentified surveys are publicly available for use at the CDC’s website. As such, investigators using BRFSS data are exempt from needing to obtain informed consent or undergoing further IRB review from their respective institution.

### Statistical analyses

We estimated the unadjusted SSD prevalence for rural and urban women during 2016–2020. We then created logistic models to determine associations between rural/urban residence status and SSD among US women with prediabetes. From these models, we obtained unadjusted odds ratios (ORs) and adjusted ORs that adjusted for age, race, education, income, health care coverage, and having a personal doctor. All statistical analyses were conducted in SAS®, version 9.4 (SAS Institute Inc, Cary NC) software and incorporated survey weighting to account for the BRFSS’s weighting scheme. Statistical testing was two-sided, determined using the Wald Chi-square value, and performed at α = 0.05.

## Results

We included 20,997 women with prediabetes in our study (Table 1). 33.7% of these women resided in rural US areas. Women with prediabetes in rural areas were comparatively more likely to be White (rural % White: 83.5%, urban % White: 66.4%), have a lower income (rural % <$25,000: 42.3%, urban % <$25,000: 30.6%), and have completed fewer years of education (rural % college graduate: 14.8%, urban % college graduate: 27.1%) than their urban counterparts.

**Table 1:** Characteristics of women with prediabetes participating in the 2016–2020 Behavioral Risk Factor Surveillance System (BRFSS) surveys.

Covariates	Rural (n = 7082)	Urban (n = 13915)
	Weighted %^1^ (95% Confidence Interval)	Weighted % (95% Confidence Interval)
**Age**		
18–44	12.7 (10.6, 14.7)	10.8 (9.6, 11.9)
45–64	41.0 (38.2, 43.9)	40.4 (38.6, 42.2)
65 and up	46.3 (43.5, 49.1)	48.8 (47.0, 50.6)
**Race**		
White	83.5 (81.7, 85.4)	66.4 (64.5, 68.3)
Black	8.9 (7.5, 10.3)	17.3 (15.9, 18.8)
Other	7.6 (6.2, 9.0)	16.3 (14.6, 18.0)
**Income**		
Less than $15,000	16.5 (14.2, 18.9)	12.0 (10.5, 13.4)
$15,000 to less than $25,000	25.8 (23.1, 28.5)	18.6 (17.1, 20.0)
$25,000 to less than $35,000	13.5 (11.4, 15.5)	11.3 (10.2, 12.3)
$35,000 to less than $50,000	14.7 (12.9, 16.5)	13.6 (12.4, 14.7)
$50,000 or more	29.5 (27.3, 31.8)	44.6 (42.8, 46.4)
**Education**		
Did not complete high school	15.7 (13.1, 18.3)	10.9 (9.2, 12.5)
High school graduate	40.1 (37.3, 43.0)	28.9 (27.3, 30.6)
Some college or technical school	29.3 (27.1, 31.5)	33.1 (31.5, 34.7)
College graduate	14.8 (13.4, 16.2)	27.1 (25.6, 28.5)
**Health insurance**		
No	5.7 (4.3, 7.1)	3.6 (2.9, 4.2)
Yes	94.3 (92.9, 95.7)	96.4 (95.8, 97.1)
**Personal doctor**		
No	8.4 (6.8, 10.1)	5.0 (4.1, 5.9)
Yes, 1	84.6 (82.2, 86.9)	86.5 (85.1, 87.8)
Yes, >1	7.0 (5.3, 8.7)	8.5 (7.4, 9.6)

^1^BRFSS weighting has been applied to calculate survey-weighted prevalences

Between 2016–2020, unadjusted SSD prevalence was found to be similar between rural (35.5%, 95% CI: 33.0%-38.0%) and urban women (35.4%, 95% CI: 33.7%-37.1%). Using unadjusted logistic regression, rural residence was not associated with SSD among US women with prediabetes (Odds Ratio (OR): 1.00, 95% CI: 0.87–1.14) (Table 2). Following adjustment for sociodemographic factors, there continued to be no association between rural residence and SSD (OR: 1.06, 95% CI: 0.92–1.22). Furthermore, among US women with prediabetes, being aged 18–44 years (OR: 2.22, 95% CI: 1.75–2.82) and aged 45–64 years (OR: 1.64, 95% CI: 1.41–1.92) was associated with significantly higher odds of short sleep duration compared to being aged 65 years and up, being Black (OR: 1.41, 95% CI: 1.17–1.70) with significantly higher odds of short sleep duration compared to being White, and earning <$50,000 with significantly higher odds of short sleep duration compared to earning ≥$50,000.

**Table 2:** Associations between rural/urban residence and short sleep duration among US women with prediabetes in the 2016–2020 Behavioral Risk Factor Surveillance System (BRFSS) surveys^2^.

Covariates	Unadjusted Model (Odds Ratio, 95% CI)	Adjusted Model (Odds Ratio, 95% CI)
**Rural/Urban Residence (reference group: Rural)**		
Urban	1.00 (0.87, 1.14)	1.06 (0.92, 1.22)
**Age (Reference group: 65 and up)**		
18–44	--	**2.22 (1.75, 2.82)**
45–64	--	**1.64 (1.41, 1.92)**
**Race (Reference** group: White)		
Black	--	**1.41 (1.17, 1.70)**
Other	--	1.16 (0.91, 1.48)
**Income (Reference group: $50,000 or more)**		
Less than $15,000	--	**1.71 (1.32, 2.20)**
$15,000 to less than $25,000	--	**1.33 (1.08, 1.63)**
$25,000 to less than $35,000	--	**1.36 (1.10, 1.69)**
$35,000 to less than $50,000	--	**1.29 (1.06, 1.57)**
**Education (Reference group: High school graduate)**		
Did not complete high school	--	**1.46 (1.10, 1.94)**
Some college or technical school	--	1.09 (0.93, 1.29)
College graduate	--	0.90 (0.75, 1.09)
**Health insurance (Reference group: Yes)**		
No	--	1.14 (0.82, 1.60)
**Personal doctor (Reference group: No)**		
Yes, 1	--	1.08 (0.79, 1.47)
Yes, >1	--	0.97 (0.66, 1.43)

^2^Bolded odds ratios indicate associations with p<0.05

## Discussion

We determined levels of SSD by rural/urban residence status in a nationally representative sample of US women with prediabetes. Regardless of residence status, around 35% of US women with prediabetes had SSD. Prevalence of and odds of SSD were similar between rural and urban women in unadjusted and adjusted analyses.

Previous US research examining SSD among individuals with prediabetes has yielded varied results [16–18]. In a study following American Indians and Alaskan natives with prediabetes between 2006–2009, 41.3% of individuals in the study self-reported having SSD (≤6 hours of sleep/night) [16]. Using National Health and Nutrition Examination Survey data from a similar time frame (2005–2008), investigators observed that 56.9% of participants in the survey slept ≤6 hours/night [17]. More contemporary research coming from the Restoring Insulin Secretion Consortium randomized controlled trials (2013–2017) conducted in Illinois, Indiana, Washington, and California found that 31.9% of participants in the trial reported sleeping <6 hours/night [18].

Differences between our findings and those from previous US research may be attributed to differing definitions of SSD and improvements in sleep duration over time as part of US preventative health initiatives (i.e., Healthy People 2020) [15]. Moreover, comparison of our inferences around sociodemographic factors with inferences from earlier studies is also challenging as those existing studies do not provide estimates of associations between sociodemographic factors and SSD [16–18]. However, our findings align with CDC estimates of SSD for the general US population showing greater levels of SSD among those aged <65 years and Blacks having the second highest SSD levels (43.5%, 95% CI: 42.3–44.6%) after Native Hawaiian/Pacific Islanders [26]. Future study examining the reasons as to why women with prediabetes from certain sociodemographic groups experience increased SSD is needed. Our study expands on prior US short sleep and prediabetes research by providing previously unknown SSD estimates for women and showing that rural women with prediabetes have SSD levels that are as high as their urban counterparts.

While our finding of similar SSD levels in rural and urban women may be somewhat surprising, it may be explained by findings from prior research on sleep disrupting factors such as noise and light pollution [27–29]. There is evidence that noise pollution does not significantly differ between rural and urban areas [27] and that some rural areas have higher levels of light pollution than urban areas [28, 29]. Thus, rural women may experience multiple factors that predispose them to SSD, potentially explaining our findings.

Although US estimates of SSD specific to women without prediabetes are unavailable, CDC statistics indicate that 32.2% (95% CI: 31.7–32.7%) of women in the general US population have SSD, which is lower that what we found for rural and urban women with prediabetes [26]. High levels of SSD among women with prediabetes present a potential opportunity to reduce prediabetes to diabetes progression. Efforts to improve SSD among rural women in medically underserved areas may be especially challenging due to access to care-related factors, such as primary care providers and sleep specialists. For example, the limited number of primary care providers in rural areas may contribute to rural residents being unaware of the risk associated with SSD and prediabetes to diabetes progression [30]. In addition, there is evidence that between 2010–2021 a lack of clinics and sleep specialists in rural areas led to lower receipt of sleep care along US rural compared to urban residents [31, 32]. Although telehealth could be a viable sleep care option for rural residents, potential obstacles include the low availability of broadband required for telehealth in rural areas and the necessity of in-person overnight sleep diagnostic tests [33, 34]. Further efforts to improve SSD among women with prediabetes, especially efforts in conjunction with improving other known diabetes risk factors, are warranted and could be instrumental in reducing the high diabetes burden in rural areas.

Our study has several limitations. Sleep duration is self-reported and, as such, it is possible there may be some self-report and non-response bias. However, studies have found that self-reported sleep duration is highly correlated with measurements from actigraphy, and self-reported sleep has been widely used in existing US research looking at SSD among people with prediabetes [16–18, 35]. Furthermore, it is likely that if any of the mentioned biases were present, they would be non-differential and lead to more conservative estimates [15]. We are unable to include SSD estimates for women with prediabetes who are institutionalized, but, as individuals who are institutionalized represent a small fraction of the US population, our findings should be generalizable to the majority of US women with prediabetes [36, 37].

## Conclusion

While SSD estimates among US women with prediabetes did not vary by rural/urban residence status, there was, nonetheless, a high level of SSD in rural areas. Additional focus on strategies to improve access to sleep care among women in rural areas, particularly those from certain sociodemographic backgrounds, in tandem with managing other diabetes risk factors may be helpful to reducing prediabetes to diabetes progression in this medically underserved high diabetes burden population.

## References

[1] Centers for Disease Control & Prevention. Diabetes Tests 2022 [February 24, 2023]. Available from: https://www.cdc.gov/diabetes/basics/getting-tested.html.

[2] Tabák AG, Herder C, Rathmann W, Brunner EJ, Kivimäki M. Prediabetes: a high-risk state for diabetes development. Lancet. 2012;379(9833):2279–90. Epub 2012/06/12. doi: 10.1016/S0140-6736(12)60283-9 ; PubMed Central PMCID: PMC3891203. 22683128 PMC3891203

[3] Centers for Disease Control & Prevention. 2020 National Diabetes Statistics Report Estimates of Diabetes and Its Burden in the United States 2020 [October 23, 2022]. Available from: https://www.cdc.gov/diabetes/pdfs/data/statistics/national-diabetes-statistics-report.pdf.

[4] Dall TM, Yang W, Gillespie K, Mocarski M, Byrne E, Cintina I, et al. The economic burden of elevated blood glucose levels in 2017: diagnosed and undiagnosed diabetes, gestational diabetes mellitus, and prediabetes. Diabetes care. 2019;42(9):1661–8. doi: 10.2337/dc18-1226 30940641 PMC6702607

[5] Centers for Disease Control and Prevention. National Diabetes Statistics Report, 2020 2020 [September 30, 2021]. Available from: https://www.cdc.gov/diabetes/data/statistics-report/index.html.

[6] Barker LE, Kirtland KA, Gregg EW, Geiss LS, Thompson TJ. Geographic distribution of diagnosed diabetes in the U.S.: a diabetes belt. Am J Prev Med. 2011;40(4):434–9. doi: 10.1016/j.amepre.2010.12.019 . 21406277

[7] Centers for Disease Control & Prevention. Prevalence of Prediabetes Among Adults 2022 [November 16, 2022]. Available from: https://www.cdc.gov/diabetes/data/statistics-report/prevalence-of-prediabetes.html.

[8] Centers for Disease Control & Prevention. Prediabetes 2022 [November 16, 2022]. Available from: https://www.cdc.gov/diabetes/library/reports/reportcard/prediabetes.html.

[9] Beulens J, Rutters F, Rydén L, Schnell O, Mellbin L, Hart HE, et al. Risk and management of pre-diabetes. Eur J Prev Cardiol. 2019;26(2_suppl):47–54. doi: 10.1177/2047487319880041 . 31766914

[10] US Department of Health and Human Services. Why Diabetes is a Concern for Rural Communities 2022 [October 4, 2022]. Available from: https://www.ruralhealthinfo.org/toolkits/diabetes/1/rural-concerns.

[11] Callaghan T, Ferdinand AO, Akinlotan MA, Towne SD Jr, Bolin J. The changing landscape of diabetes mortality in the United States across region and rurality, 1999‐2016. The Journal of Rural Health. 2020;36(3):410–5. doi: 10.1111/jrh.12354 30802321

[12] Befort CA, Nazir N, Perri MG. Prevalence of obesity among adults from rural and urban areas of the United States: findings from NHANES (2005‐2008). The Journal of Rural Health. 2012;28(4):392–7. doi: 10.1111/j.1748-0361.2012.00411.x 23083085 PMC3481194

[13] Mostafa SA, Mena SC, Antza C, Balanos G, Nirantharakumar K, Tahrani AA. Sleep behaviours and associated habits and the progression of pre-diabetes to type 2 diabetes mellitus in adults: A systematic review and meta-analysis. Diab Vasc Dis Res. 2022;19(3):14791641221088824. Epub 2022/05/27. doi: 10.1177/14791641221088824 ; PubMed Central PMCID: PMC9152198. 35616501 PMC9152198

[14] Rooney MR, Rawlings AM, Pankow JS, Echouffo Tcheugui JB, Coresh J, Sharrett AR, et al. Risk of Progression to Diabetes Among Older Adults With Prediabetes. JAMA Internal Medicine. 2021;181(4):511–9. doi: 10.1001/jamainternmed.2020.8774 33555311 PMC7871207

[15] Matthews KA, Croft JB, Liu Y, Lu H, Kanny D, Wheaton AG, et al. Health-Related Behaviors by Urban-Rural County Classification—United States, 2013. MMWR Surveill Summ. 2017;66(5):1–8. Epub 20170203. doi: 10.15585/mmwr.ss6605a1 ; PubMed Central PMCID: PMC5829834. 28151923 PMC5829834

[16] Nuyujukian DS, Beals J, Huang H, Johnson A, Bullock A, Manson SM, et al. Sleep duration and diabetes risk in American Indian and Alaska native participants of a lifestyle intervention project. Sleep. 2016;39(11):1919–26. doi: 10.5665/sleep.6216 27450685 PMC5070746

[17] Engeda J, Mezuk B, Ratliff S, Ning Y. Association between duration and quality of sleep and the risk of pre-diabetes: evidence from NHANES. Diabet Med. 2013;30(6):676–80. Epub 2013/02/22. doi: 10.1111/dme.12165 ; PubMed Central PMCID: PMC3660430. 23425048 PMC3660430

[18] Mokhlesi B, Temple KA, Tjaden AH, Edelstein SL, Utzschneider KM, Nadeau KJ, et al. Association of Self-Reported Sleep and Circadian Measures With Glycemia in Adults With Prediabetes or Recently Diagnosed Untreated Type 2 Diabetes. Diabetes Care. 2019;42(7):1326–32. Epub 2019/05/03. doi: 10.2337/dc19-0298 ; PubMed Central PMCID: PMC6609965. 31048411 PMC6609965

[19] Centers for Disease Control & Prevention. About BRFSS 2014 [August 15, 2022]. Available from: https://www.cdc.gov/brfss/about/index.htm.

[20] Kuehn BM. Sleep Duration Linked to Cardiovascular Disease. Circulation. 2019;139(21):2483–4. doi: 10.1161/CIRCULATIONAHA.119.041278 31107620

[21] Centers for Disease Control and Prevention. LLCP 2019 Codebook Report Overall version data weighted with _LLCPWT Behavioral Risk Factor Surveillance System 2020. Available from: https://www.cdc.gov/brfss/annual_data/2019/pdf/codebook19_llcp-v2-508.HTML.

[22] Centers for Disease Control and Prevention. LLCP 2017 Codebook Report Overall version data weighted with _LLCPWT Behavioral Risk Factor Surveillance System 2018 [October 21, 2022]. Available from: https://www.cdc.gov/brfss/annual_data/2017/pdf/codebook17_llcp-v2-508.pdf.

[23] Centers for Disease Control & Prevention. LLCP 2016 Codebook Report Overall version data weighted with _LLCPWT Behavioral Risk Factor Surveillance System 2016 [November 16, 2022]. Available from: https://www.cdc.gov/brfss/annual_data/2016/pdf/codebook16_llcp.pdf.

[24] Centers for Disease Control & Prevention. LLCP 2018 Codebook Report Overall version data weighted with _LLCPWT Behavioral Risk Factor Surveillance System 2019 [November 16, 2022]. Available from: https://www.cdc.gov/brfss/annual_data/2018/pdf/codebook18_llcp-v2-508.pdf.

[25] Centers for Disease Control & Prevention. LLCP 2020 Codebook Report Overall version data weighted with _LLCPWT Behavioral Risk Factor Surveillance System 2021 [Novmeber 16, 2022]. Available from: https://www.cdc.gov/brfss/annual_data/2020/pdf/codebook20_llcp-v2-508.pdf.

[26] Centers for Disease Control & Prevention. Sleep and Sleep Disorders 2022 [February 24, 2023]. Available from: https://www.cdc.gov/sleep/data-and-statistics/adults.html.

[27] Casey JA, Morello-Frosch R, Mennitt DJ, Fristrup K, Ogburn EL, James P. Race/ethnicity, socioeconomic status, residential segregation, and spatial variation in noise exposure in the contiguous United States. Environ Health Perspect. 2017;125(7):077017. doi: 10.1289/EHP898 28749369 PMC5744659

[28] Cox D, de Miguel AS, Bennie J, Dzurjak S, Gaston K. Majority of artificially lit Earth surface associated with the non-urban population. Science of The Total Environment. 2022;841:156782. doi: 10.1016/j.scitotenv.2022.156782 35724779

[29] Falchi F, Furgoni R, Gallaway TA, Rybnikova NA, Portnov BA, Baugh K, et al. Light pollution in USA and Europe: The good, the bad and the ugly. Journal of environmental management. 2019;248:109227. doi: 10.1016/j.jenvman.2019.06.128 31362173

[30] Nielsen M, D’Agostino D, Gregory P. Addressing Rural Health Challenges Head On. Mo Med. 2017;114(5):363–6. ; PubMed Central PMCID: PMC6140198. 30228634 PMC6140198

[31] Chang JJ, Salas J, Habicht K, Pien GW, Stamatakis KA, Brownson RC. The association of sleep duration and depressive symptoms in rural communities of Missouri, Tennessee, and Arkansas. J Rural Health. 2012;28(3):268–76. Epub 2012/07/05. doi: 10.1111/j.1748-0361.2011.00398.x ; PubMed Central PMCID: PMC3476945. 22757951 PMC3476945

[32] Folmer RL, Smith CJ, Boudreau EA, Totten AM, Chilakamarri P, Atwood CW, et al. Sleep disorders among rural Veterans: Relative prevalence, comorbidities, and comparisons with urban Veterans. J Rural Health. 2022. Epub 20221105. doi: 10.1111/jrh.12722 . 36333991

[33] Spagnuolo CM, McIsaac M, Dosman J, Karunanayake C, Pahwa P, Pickett W. Distance to Specialist Medical Care and Diagnosis of Obstructive Sleep Apnea in Rural Saskatchewan. Can Respir J. 2019;2019:1683124. Epub 20190114. doi: 10.1155/2019/1683124 ; PubMed Central PMCID: PMC6348862. 30733845 PMC6348862

[34] Graves JM, Abshire DA, Amiri S, Mackelprang JL. Disparities in Technology and Broadband Internet Access Across Rurality: Implications for Health and Education. Fam Community Health. 2021;44(4):257–65. doi: 10.1097/FCH.0000000000000306 ; PubMed Central PMCID: PMC8373718. 34269696 PMC8373718

[35] Kohatsu ND, Tsai R, Young T, Vangilder R, Burmeister LF, Stromquist AM, et al. Sleep duration and body mass index in a rural population. Arch Intern Med. 2006;166(16):1701–5. Epub 2006/09/20. doi: 10.1001/archinte.166.16.1701 . 16983047

[36] Harris-Kojetin LD, Sengupta M, Lendon JP, Rome V, Valverde R, Caffrey C. Long-term care providers and services users in the United States, 2015–2016. 2019. 27023287

[37] Carson EA. Prisoners in 2020–Statistical tables. NCJ. 2021;302776:1–50.

